# Supplementary material for: Complexome profiling of the Chlamydomonas psb28 mutant reveals TEF5 as an early PSII assembly factor
Source: Plant Cell. 2025 Mar 17;37(6):koaf055. doi: 10.1093/plcell/koaf055 (PMC12164590; doi:10.1093/plcell/koaf055)
Supplement: koaf055_Supplementary_Data [file koaf055_supplementary_data.zip › psb28_interactive.zip!/plots/intensity/qualityPlots/peptides/_Cre02.g073550.t1.2.html]

### Cre02.g073550.t1.2

Trivial name:   
SeqCoverage [median]: 9.40  
Mass: 39.40 kDa  
  
 

| Sequence | MissedCleavage | Mass [Da] | wtIntensity | muIntensity | Score |
| --- | --- | --- | --- | --- | --- |
| DVTVEEQK | 0 | 946.46 | 271.21 | 0.00 | 87.32 |
| EYQAELAQLQSK | 0 | 1406.70 | 0.00 | 928.05 | 82.49 |
| VYVLEPEDDVVPK | 0 | 1500.77 | 387.45 | 1497.26 | 77.68 |
|  |  |  | 658.7 | 2425.3 |  |
